# The Impact of Seasonality on Mental Health Disorders: A Narrative Review and Extension of the Immunoseasonal Theory

**DOI:** 10.3390/jcm14041119

**Published:** 2025-02-09

**Authors:** Stefan Modzelewski, Maciej Naumowicz, Maria Suprunowicz, Aleksandra Julia Oracz, Napoleon Waszkiewicz

**Affiliations:** Department of Psychiatry, Medical University of Bialystok, pl. Wołodyjowskiego 2, 15-272 Białystok, Poland; 38741@student.umb.edu.pl (M.N.); maria.suprunowicz@sd.umb.edu.pl (M.S.); aleksandra.oracz@sd.umb.edu.pl (A.J.O.); napoleon.waszkiewicz@umb.edu.pl (N.W.)

**Keywords:** immunoseasonal, weather, inflammation, depression, mental disorders, schizophrenia, bipolar disorder

## Abstract

The impact of weather on mental illness is widely debated, but the mechanism of this relationship remains unclear. The immunoseasonal theory suggests that in winter, a T-helper 1 (Th1) response predominates, impairing Prefrontal Cortex (PFC) control, which exacerbates symptoms of depression, while after it, in summer, a Th2 response predominates in immunologically prone individuals, activating cortical and mesolimbic centers, which can exacerbate symptoms of psychosis. In this paper, we aim to describe the validity of this theory through a narrative review of data related to weather and immunology in psychiatry. This review extends existing literature by integrating immunological findings with psychiatric seasonality research, offering a mechanistic perspective that links Th1/Th2 shifts to specific symptom exacerbations. Winter Th1 severity may worsen depression and anxiety, while summer Th2 dominance appears to be associated with exacerbations of schizophrenia, mania, impulsivity, and suicide risk. It is possible that the mechanism of Th1 response potentiation and deterioration of PFC function is common to most psychiatric entities and is nonspecific. This suggests that seasonal immune dysregulation may play a broader role in psychiatric disorders than previously recognized, challenging the idea that seasonality impacts only selected conditions. Characteristic dysfunctions within an individual determine further differences in clinical manifestations. The mechanism of Th2 potentiation may not be limited to mania and psychosis but may also be associated with increased impulsivity and suicide risk. If the immunoseasonal theory is confirmed, selected immunological markers could be used not only in the diagnosis of psychiatric exacerbations but also in predicting symptom fluctuations and tailoring treatment strategies. This could enable more personalized interventions, such as seasonally adjusted medication dosing or targeted anti-inflammatory therapies. While this mechanism seems plausible, further research, especially analyzing markers of inflammatory and anti-inflammatory responses, is needed to better understand and confirm it.

## 1. Introduction

The influence of weather and climate on the course of mental illnesses has, for a long time, been of interest to researchers. Preliminary data indicate that certain weather patterns, periods of autumn-winter or early spring, are associated with exacerbation of symptoms of mental illnesses, such as depression, schizophrenia, manic disorders, or anxiety disorders [1]. Despite promising research results, the pathway through which weather would affect the symptoms and course of mental illnesses remains unclear. It is possible that changes in individual weather components, such as humidity or sunlight, are involved in the mechanism [2]. One of the existing hypotheses addressing this issue is the Circadian Hypothesis of Depression, which postulates that changes in depression symptomatology are linked to the biological rhythm of the body. Interestingly, researchers have also identified diurnal mood variations in healthy individuals, suggesting the presence of a general mechanism that, in the case of depression, may be pathologically altered yet still observable. Its implications for seasonality could be explained through the modulation of light intensity across different seasons, as well as fluctuations in the duration and concentration of melatonin—a key regulator of circadian rhythms [3]. However, despite empirical evidence, such as the therapeutic effects of light therapy, studies investigating specific biomarkers (e.g., melatonin levels) remain inconclusive [4,5].

The immunoseasonal theory of mental illness proposes that exacerbations of psychiatric symptoms at certain times of the year are the result of seasonal changes in immune system activity, particularly the balance between Th1 and Th2 responses. The Th1 response, associated with pro-inflammatory processes, can exacerbate depressive symptoms by increasing the activity of inflammatory markers such as IL-1, IL-6, and TNF-α [6]. In contrast, the Th2 response, specific to anti-inflammatory responses, may be associated with the development of psychotic symptoms, particularly in the context of schizophrenia [7]. Moreover, the immunoseasonal hypothesis provides a framework for integrating previous findings suggesting that biological rhythms may be influenced by dysfunctions in cortical and subcortical centers resulting from inflammation (a more detailed description of the presumed mechanism is described further in the text).

The aim of this study is to evaluate the impact of seasonality on the exacerbations of psychiatric symptoms in the context of the immunoseasonal theory. We will attempt to gather evidence that observatively and retrospectively points to exacerbations during the seasons indicated by the theory. To better understand the weather conditions under which exacerbations may occur, it is necessary to characterize the various components of the weather. We will evaluate the information data in the context of potential immune mechanisms studied in mental illness.

## 2. Materials and Methods

Since our study is a narrative review, we did not apply strictly defined inclusion criteria. We included articles that met a few essential criteria: they were original research studies, including observational, cross-sectional, and retrospective studies, or comprehensive systematic reviews and meta-analyses. These articles focused on topics related to mental disorders associated with weather factors, seasonality, and cytokine imbalances in psychiatric disorders. We included studies from various geographical regions to provide a broader description of the data. Additionally, we included literature that supports the immunoseasonal theory. Exclusion criteria included studies not available in English to minimize the risk of translation errors and misinterpretation, as well as to ensure that the source literature remains accessible to the majority of readers in its original form. Case reports and case series were excluded as they do not allow for generalizable conclusions regarding seasonality and immunological mechanisms. Commentaries, letters to the editor, and reviews without a defined methodology were also not included. Studies conducted exclusively on pediatric or geriatric populations were excluded unless they provided comparative data with adult patients. Research focusing on psychiatric disorders with a primarily organic etiology (e.g., dementia, neurological conditions) was not considered, as their pathophysiology differs from that of non-organic psychiatric disorders. PubMed and Web of Science databases were searched using the following terms: ‘biomarkers’, ‘psychiatry’, ‘cytokine’ ‘inflammation’, ‘psychosis’, ‘mania‘, ‘schizophrenia’, ‘depression’, ‘bipolar disorder’, ‘seasonal’, ‘weather’, ‘winter’, ‘autumn’, ‘summer’, ‘spring’, ‘atmospheric pressure’, ‘sunlight’, ‘wind’, ‘humidity’, ‘temperature’ as well as variations and combinations of these terms. Given that this study is a narrative review, a formal systematic quality assessment tool was not applied. However, studies were selected based on their relevance to the aim of the study, methodological rigor, and contribution to the understanding of seasonality in psychiatric disorders. Priority was given to studies with well-defined methodologies, representative sample sizes, and objective measurements of immunological and meteorological variables. Additionally, preference was given to peer-reviewed articles published in reputable journals to ensure the reliability of the included literature. 

## 3. Immunological Mechanisms Linked to Seasonality: Inflammatory Markers in Mental Disorders

### 3.1. Background

**Figure 1:**
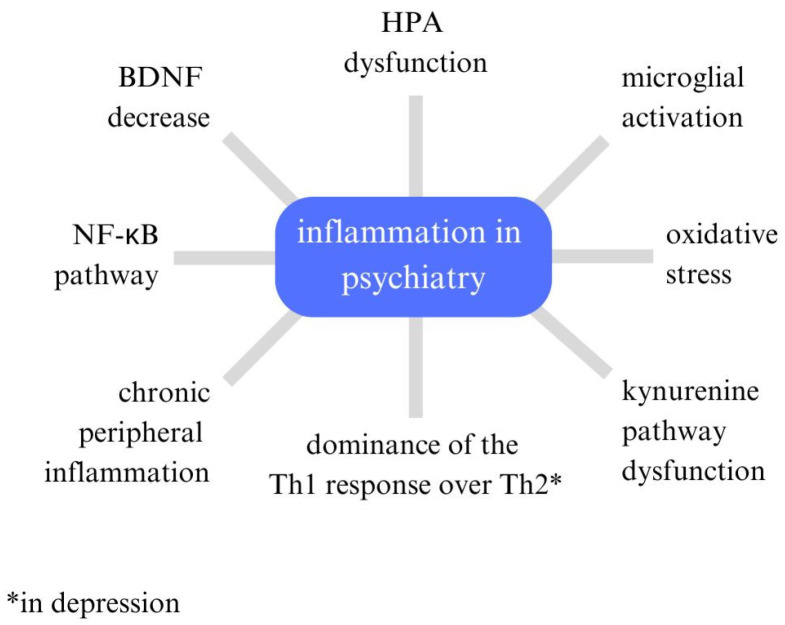
Current factors linked to inflammation in psychiatry.

The connection between the weather, its factors, and the mental state of psychiatric patients has recently been gaining popularity, taking into account, among other things, climate change [8], but the mechanisms that explain how weather affects the mental state of psychiatric patients are not known. Immune mechanisms may be an attempt to answer. In the history of Psychiatry research, the immune system remains one of the lines that may be implicated in the pathogenesis of psychiatric disorders [9,10,11]. In the context of today’s discoveries, mechanisms are proposed through which systemic inflammation may have its outcome in the central nervous system (CNS). In this case, relevant are the mechanisms that happen on both sides of the blood-brain barrier: on the one hand, there is an increased activation of microglia, and astrocytes, which produce denovo pro-inflammatory cytokines, and there may be an increased activation of the kineurin pathway [12], the equivalent of which may be reduced serotonin production from tryptophan, further pro-inflammatory effects, and inhibition of brain-derived neurotrophic factor (BDNF) in the brain. The situation in the CNS can lead to the unsealing of the blood-brain barrier. In addition, immune T cells, in response to the ongoing inflammatory process peripherally, produce adhesins that bind to the surface of the blood–brain barrier (BBB), paving the way for pro-inflammatory cytokines, as observed in neurological diseases, among others [13,14]. Peripheral cytokines through BBB penetration act by activating cyclooxygenase-2 (COX-2) and increased production of prostaglandin E2 (PGE2) and nitric oxide (NO) [15]. In addition, cytokines can stimulate wall macrophages, microglia, and astrocytes to secrete more cytokines. This process also decreases BDNF levels and activates the enzyme indoleamine 2,3-dioxygenase (IDO), which in turn enhances excitotoxicity. The whole neuroinflammation may be further potentiated by Hypothalamic–pituitary–adrenal (HPA) axis dysfunction, which, in the case of mental illnesses (mainly affective), may, as a result of the process of chronic hyperkinesia, further damage hypothalamic structures [16]. In the case of schizophrenia, on the other hand, prolonged psychosis is a process leading to CNS neurodegeneration, probably due to glutaminergic exotoxicity, which is also mediated by pro-inflammatory cytokines [17,18,19] (Figure 1).

### 3.2. Depression

In the case of depression, Maes et al. showed increased proliferative activity of lymphocytes, indicating an important role for such cytokines as IFN-γ, Il-1β, and IL-6. This stands in line with information from Smith’s macrophage theory of depression in which he identified, in addition to il-1β, IFN-α, and TNF-α among the main substances that can provoke and influence the course of depression [20]. In addition, these data were in partial agreement with those from cerebrospinal fluid (CSF) [21], and higher cytokine values were found in patients with drug-resistant depression [22]. In addition, later studies also indicated the normalization of inflammatory parameters with clinical improvement [23]. Additionally, in the case of infection studies, more severe inflammation at admission indicated higher scores on depression scales [24], which is consistent with Maes’ theory that puts inflammation as one of the main determinants of the course of depression [9]. Many other studies point to an important etiological role of the inflammatory component in depression [15,25]. Previous meta-analyses specifically point to the contribution of substances such as the aforementioned Il-6, TNF-α [26], and CRP [27].

### 3.3. Schizophrenia

In schizophrenia, the inflammatory component is not as well-established as it is in depression. However, a significant pro-inflammatory response is often observed during the first episode of psychosis. Studies have shown that first-episode psychosis is associated with elevated levels of blood cytokines, including IL-1β, sIL2R, IL-6, TNF-α, and TGFβ, as well as an increase in total lymphocyte count [17,28]. Further evidence supports an altered immune response in schizophrenia, with interleukins like IL-6 and IFN-γ being notably affected [29]. In acute schizophrenia-spectrum disorders, IL-2 and IFN-γ levels tend to be significantly elevated, while in chronic cases, IL-4, IL-12, and IFN-γ levels are often decreased, indicating distinct immunological profiles across different stages of the disorder [30].

### 3.4. Bipolar Disorder

Bipolar disorder (BP) is a disorder with an incompletely understood etiology. The development of immunological theories has also led to the analysis of this entity in relation to the others. BP is a disease that has a higher incidence of autoimmune diseases, such as autoimmune hepatitis, Guillain-Barre syndrome, Crohn’s disease, and systemic lupus erythematosus [31]. In a comparison of bipolar disorder patients in manic and depressive phases to healthy controls, a pro-inflammatory profile is observed. This includes elevated levels of pro-inflammatory cytokines and reduced levels of anti-inflammatory cytokines, indicating an ongoing inflammatory imbalance across mood states. During euthymia, there is no elevation of inflammatory parameters [32]. Viewed as a whole, a state of immune system activation (such as TNF-α, CRP, Il-6) is observed in BP, which is evident at disease exacerbation, whether it is a manic or depressive episode [33,34,35,36]. Moreover, selected data indicate that IL-6 seems to be the predominant cytokine, IL-4 dominates in the euthymic state, and IL-2, IL-4, and IL-6 are prevalent in the manic state [37].

### 3.5. Generalized Anxiety Disorder (GAD)

Chronic stress, as seen in conditions like generalized anxiety disorder (GAD) and depression, can lead to dysfunction of the hypothalamic-pituitary-adrenal (HPA) axis, prolonged sympathomimetic activation, and a paradoxical enhancement of immune activity [38]. This response likely involves a dual mechanism: on one hand, HPA activation can suppress immunity, increasing susceptibility to infections; on the other hand, it can mask typical infection symptoms, leading to a so-called “silent infection”. Moreover, prolonged HPA activation does not entirely inhibit the release of certain inflammatory factors, as indicated by Smith et al. [20]. Research on inflammatory markers in GAD has shown elevated levels of pro-inflammatory cytokines, such as TNF-α and C-reactive protein, as well as an increased TH17 response. Conversely, anti-inflammatory markers like IL-4 are often reduced in GAD patients, supporting the theory that chronic stress in GAD contributes to an inflammatory state [39,40,41].

### 3.6. PTSD

The mechanisms linking trauma, stress, and inflammation are particularly pronounced in PTSD, where they can manifest acutely and with significant intensity. Research by Passos et al. (2015) demonstrated that individuals with PTSD had elevated markers of pro-inflammatory response, including increased levels of interleukin-6 (IL-6), interleukin-1β (IL-1β), TNF-α, and interferon-γ [42,43,44]. Additionally, symptom severity in PTSD patients has been found to correlate with elevated IL-6 levels, suggesting a potential link between inflammation and the clinical severity of PTSD symptoms [42].

### 3.7. ASD

Genetic and environmental factors have been attributed to the pathogenesis of autism spectrum disorder [45]. Numerous studies also suggest a role of abnormal immune system function, especially an increase in pro-inflammatory Th1 response cytokines such as IFN-γ, IL-1β, IL-6, and TNF-α [46,47]. In contrast, the levels of cytokines specific to the Th2 response, namely, IL-4, IL-5, IL-10, and IL-13, appear to be unchanged in individuals with ASD [48]. Although some researchers find no evidence of inflammation in individuals with autism [49], a meta-analysis based on 37 cytokines from 289 studies provides evidence for an abnormal cytokine profile in ASD, showing that IL-6, IL-1β, IL-12p70, macrophage migration inhibitory factor (MIF), eotaxin-1, monocyte chemotactic protein-1 (MCP-1), IL-8, IL-7, IL-2, IL-12, tumor necrosis factor-α (TNF-α), IL-17 and IL-4 are elevated in autism [47]. Dysregulation of pro-inflammatory and regulatory T cells may also have an impact on the development and course of autism, with lower levels of regulatory T cells and higher levels of pro-inflammatory Th17 cells observed in ASD patients [50]. A link between inflammation and the severity of autism and greater impairment of abnormal behavior has also been suggested. Rossi et al. showed that children diagnosed with ASD whose plasma reacted with brain tissue with present autoantibodies had increased behavioral and emotional problems [51]. Behavioral deterioration in autistic patients may also be influenced not only by the presence of autoantibodies but also by an increase in pro-inflammatory interleukins such as IL-1β, IL-6, IL-8, and IL-12 [52].

## 4. Immunological Mechanisms Linked to Seasonality: A Pathway Between Weather and Immune System

### 4.1. Outline of the Immunoseasonal Theory

**Figure 2:**
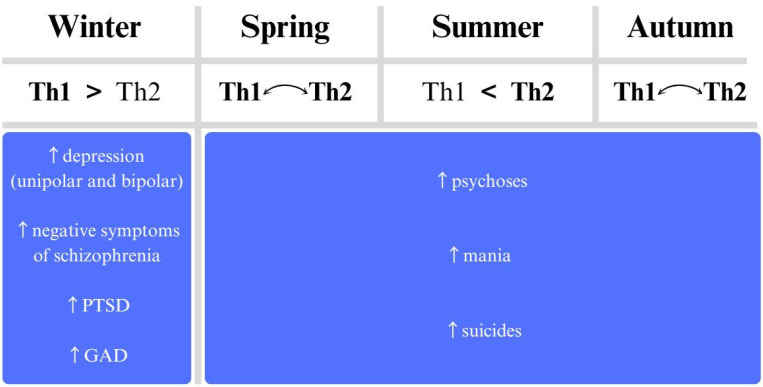
Exacerbations of mental illnesses according to the season extended to theoretical changes in Th1 and Th2 activation.

Systemic inflammation can significantly impact CNS function, a connection that supports the immune-seasonality theory of mental illness. This theory suggests that seasonal changes can influence mental health through immune system activation, specifically through Th1 and Th2 immune responses, known as the immunoseasonal theory. The Th1 response—often observed in studies of depression—is cellular and pro-inflammatory, while the Th2 response is humoral and associated with allergic reactions. Macrophages play key roles in both responses, and their involvement has been linked to the development of mood disorders such as depression and bipolar disorder (BP), as well as schizophrenia [20,53]. Th1 lymphocytes produce cytokines like IL-1β, IL-2, IL-6, TNF-α, and IFN-γ. In contrast, Th2 lymphocytes produce interleukins IL-4, IL-5, IL-10, IL-13, and transforming growth factor β (TGF-β) [54]. The hypothesis is that during certain seasonal periods, which may predispose individuals to infections, there can be an exacerbation of the chronic inflammation observed in conditions like depression and BP, potentially intensifying symptoms (Figure 2).

Viruses such as noroviruses are small enough to be transmitted through droplets, particularly when increased humidity facilitates their spread, leading to infections that activate the Th1 response [6]. These conditions are more common in winter, early autumn, and early spring [2,55,56]. However, it’s important to note that not every virus or microorganism spreads more easily in high humidity and elevated temperatures [57]. In addition, rapid weather fluctuations may have a more pronounced impact on human health by exacerbating diseases in a way that is less predictable compared to the typical seasonal cycle [58,59]. This suggests that rather than gradual seasonal changes, abrupt weather shifts can contribute to increased viral transmission and pathogen spread, influencing seasonality-related disease patterns. Moreover, the concept of seasonality, where viral infections are reduced during the summer months, is supported not only by empirical observations but also by studies on inflammatory response markers. For instance, markers such as IFN-γ (which is elevated during viral infections in patients with neurological disorders) and IL-6 provide additional evidence for the seasonal modulation of immune responses [59,60,61]. During specific weather conditions typically observed in winter and autumn-spring seasons, an increase in pathogen virulence may occur, which can lead to an intensified Th1 cellular response. This may result in a Th1 predominance over Th2 and a reduction in prefrontal cortex (PFC) activation, ultimately lowering mood and subsequent activity of the subcortical structures [62,63]. In cases of exacerbated positive symptoms in schizophrenia, the theory links this to a Th2 response. Th2 is typically involved in parasitic diseases and allergies, which tend to worsen during late spring and in summer. According to this theory, psychotic exacerbations are more likely to occur during these periods [6]. Schizophrenia is associated with a higher incidence of allergies and increased levels of antiparasitic antibodies [7,64].

Summer or conditions with increased allergen release may further exacerbate the Th1–Th2 imbalance towards an anti-inflammatory Th2 response in individuals immunologically prone to schizophrenia. Th2 response can lead to multifaceted cortical and mesolimbic stimulation (via cytokine pathways, interactions with the NMDA receptor, excessive glutamatergic stimulation, etc.) [65]. As Waszkiewicz suggests, psychotic mania Th2 immune states exacerbations are also more likely to occur during summer, as a response to previous Th1 hyperactivity [6].

### 4.2. Extension of the Immunoseasonal Theory

The immunoseasonal theory initially aimed to explain three major disorders: bipolar disorder (BD), schizophrenia, and unipolar depression. However, as noted above, given the systemic inflammation observed in other psychiatric disorders, the theory could be extended to include other disorders, such as generalized anxiety disorder (GAD), autism spectrum disorder (ASD), and PTSD due to overlapping symptoms. GAD and anxiety disorders are among the most common comorbidities in depression [66], and the etiology of both disorders emphasizes the role of the HPA axis. Prolonged HPA axis activation in response to chronic stress may lead to glucocorticoid resistance, potentially causing a disinhibition of the inflammatory response in GAD [38,67]. Loss of cortisol’s inhibitory role, e.g., on IL-6, may promote further inflammation through oxidative stress and activation of other cytokines [68]. Moreover, both GAD and depression show impairments in PFC function [69,70,71]. Considering the proinflammatory nature of GAD, additional exacerbating factors, such as colds, may intensify systemic inflammation, promoting a Th1 response in the CNS. This Th1 response, impairing PFC function, could further diminish its control over subcortical centers, such as the amygdala, and intensify default network activity, leading to anxiety exacerbation (loss of amygdala inhibition) and increased rumination (default network), worsening anxiety disorders [72,73].

In PTSD, inflammation has been associated with increased reactivity to stressful events [43], and patients with PTSD typically show higher levels of pro-inflammatory cytokines [74]. Additionally, the role of the HPA axis in the etiology of PTSD is emphasized [75,76,77]. In both PTSD and MDD, studies have observed hippocampal volume reduction [78,79,80], and PFC dysfunction leads to hyperactivity in regions like the amygdala, increasing tension, anxiety [81,82], and excessive reactivity [83,84]. The empirical effectiveness of SSRIs for both MDD and PTSD provides further evidence of similar pathophysiological pathways. Thus, like MDD, infectious-prone conditions may disrupt PFC control over the amygdala and hippocampus, inducing exacerbation of PTSD symptoms.

In ASD, cortical changes are heterogeneous due to its clinical classification. Unlike other disorders, ASD shows early prefrontal cortex overgrowth with structural abnormalities and a pro-inflammatory profile [85]. Some studies indicate that local functional connectivity was atypically increased in individuals with ASD during exacerbations [86], suggesting a similar pathway for PFC function deterioration as seen in depression under seasonal weather changes. However, it is important to note that ASD also involves the amygdala and default network changes, cerebellar hypoplasia, and may align with pathways more typical of schizophrenia [87].

## 5. Empirical Evidence: Characteristics of Weather Factors

### 5.1. Humidity

Humidity is a factor that correlates particularly strongly with temperature in the context of mental health. Higher temperatures promote higher humidity, raising the risk of psychiatric problems [88,89]. This may additionally be more pronounced in larger cities where there are often weaker winds [90].

Studies show that relative humidity can exacerbate the course of depression [2]. Moreover, the relative humidity, ambient temperature, and air pressure in the summer (so higher values relative to the rest of the year) may be the cause of lower binding of blood platelets to serotonin in people suffering from depression [91]. Also in the case of l-tryptofan availability, these factors—the relative humidity, ambient temperature, and air pressure—may be responsible for a decrease in its availability, which could theoretically translate into decreased serotonin synthesis [92], There are, however, studies indicating that the lower humidity, occurring a week before hospitalization, may be a risk factor for exacerbation of affective diseases [93].

Humidity remains, along with temperature, one of the more widely analyzed weather factors. However, its influence, especially in the summer season, along with high temperatures, seems to be of greater importance compared to the effect of a single factor [94].

### 5.2. Temperature

Research indicates that temperature may be one of the most significant weather factors influencing exacerbations of mental illness [95]. Studies commonly link high temperatures, or elevated air temperature, with increased rates of emergency room visits [96,97]. On dry, warm days, high temperatures appear to increase the likelihood of risky behavior and admissions to psychiatric facilities [98].

Bipolar disorder has been noted to show an increased incidence of hospitalizations during the summer months, particularly when air temperatures are higher [99]. However, findings are not consistent across studies; one investigation into mania-related hospitalizations observed a negative temperature correlation with hospitalizations of patients with BP [100] conducted in Saudi Arabia. For unipolar depressive disorder, some research suggests that rising temperatures are more strongly associated with violent suicides [101] and suicide attempts [102]. This association appears more pronounced among men [103], possibly due to suicide epidemiology.

Overall, studies suggest that high air temperature, especially on hot, dry days, may be a significant factor in mental illness exacerbations, increasing the risk of hospitalization and risky behaviors. Although findings are inconclusive, elevated temperatures may correlate with both an increase in hospitalizations for mania and higher suicide rates, particularly among men.

### 5.3. Atmospheric Pressure

Another factor to consider is barometric pressure. Compared to temperature or sunlight exposure, barometric pressure tends to remain more stable. While temperature increases in the summer can negatively impact general well-being, during the autumn, warmer temperatures are often perceived positively. By contrast, our physiological response to barometric pressure appears more predictable and less influenced by seasonal or demographic variations [104].

Barometric pressure also correlates with impulsive behaviors [105], including suicide [106,107]. Although the association is statistically small, it remains significant. Low barometric pressure is linked to days with higher suicide rates and more variable behavior, particularly among men [104]. However, the significance of the association between low pressure and suicide rates is limited by challenges in isolating the influence of weather from other suicide risk factors [108].

In some studies, lower atmospheric pressure has been associated with an increased incidence of hospitalization for schizophrenia [109]. Atmospheric pressure is often studied as part of a broader set of weather factors, where multiple elements are analyzed together [110]. Alongside humidity, barometric pressure has been investigated as a potential risk factor for depression and mania [111], although its effect on manic exacerbations remains unclear [112]. Interestingly, research on rats has shown that low atmospheric pressure can increase emotional impairment [113].

In affective disorders, other weather variables appear more strongly linked to hospitalization risk [114]. However, considering both diagnosed and undiagnosed cases, low atmospheric pressure is associated with an increased likelihood of suicide attempts, often connected with depressive symptoms.

The presented data suggest that lowering atmospheric pressure may heighten the risk of impulsive behaviors, potentially leading to suicide attempts. In schizophrenia, it may also contribute to a higher frequency of hospitalizations. The shift is small and visible despite the fact that the study collects data from different geographical latitudes. Their presence limits the results of the study [108] due to the difficulty of assessing the phenomenon in separation from the others.

### 5.4. Wind

The wind is a factor that results from differences in atmospheric pressure, which can be of particular importance due to, among other things, the transmission of vectors of viral and other infections [115,116]. In the case of exposure to wind, it intensifies energy consumption for thermoregulation. Determining its impact is all the more difficult because of reduced exposure to this factor in urban areas. It may also be relevant whether one is dealing with a mountain wind, such as fohn [117,118], or a cold wind. The atmospheric pressure changes associated with wind movements, especially those characterized by infrasound low frequency sound waves, may result in deterioration of mental state such as chronic noise stress, anxiety, depression, cognitive dysfunction, and disrupted sleep [119].

Few papers show a clear effect of wind as an isolated factor affecting exacerbations of mental illness [120]; data suggest that the lack of wind promotes hospitalization of people with depression [121], or along with moderate humidity [122]. On the other hand, in winter, with high humidity and snowfall, increasing air speed increases the risk of admission for depression [123].

### 5.5. Insolation

Insolation is also a weather factor that can affect the severity of mental illness symptoms. In assessing its importance, it is needed to consider that periodic changes in insolation may co-occur with other periodic changes in climatic factors, such as air temperature. The limiting factor in many studies of the impact of insolation is the lack of data on the level of exposure of subjects to sunlight. Most studies use parameters such as the duration of sunshine per day or the intensity of sunlight.

It is believed that insolation is one of the factors responsible for the seasonality of suicides. A weak but highly significant positive correlation is observed between the duration of sunshine and the number of suicides on the same days. It has been shown that a longer average sunshine duration in the days preceding a suicide (up to 11 days) can also influence the increased risk of suicide [124]. A study conducted in South Korea, which adjusted for the impact of other climatic variables related to the suicide rate, found that an increase in solar radiation by 1 MJ/m^2^ leads to a 1.008-fold increase in the suicide rate [125]. Additionally, high levels of solar radiation are associated with an increased rate of high-lethality suicide attempts (HLSA) [103].

On the other hand, decreased sunlight exposure has been linked to an increased risk of depression [126] and may also be associated with a higher rate of suicides [127].

Papadopoulos et al. suggest that sunlight may exert a natural antidepressant effect, initially enhancing motivation, which could transiently elevate suicide risk before subsequently improving mood [128]. This observation may also explain why patients with depression hospitalized in south-facing rooms, which have greater sunlight exposure, experience shorter hospitalizations than those in northwest-facing rooms [129].

Further research has indicated that a significant increase in sunlight exposure during spring may correlate with an earlier age of onset of bipolar disorder and an increased frequency of hospital admissions for individuals with this diagnosis [130,131,132].

It has also been observed that the high variability of sunshine between winter and summer (which is associated with closer proximity to the equator) may influence the increased number of suicide attempts in patients with bipolar disorder [133].

The response to changes in sunlight intensity, as indicated by hospitalization rates, appears to vary significantly by gender and age in patients with bipolar disorder or a single depressive episode [134].

Based on current data, it appears that during the initial period of increasing sunlight, there may be a heightened risk of impulsive behaviors, such as suicide, followed by a subsequent alleviation of depressive symptoms. Increased sunlight exposure may also be linked to an earlier onset of bipolar disorder and a rise in related hospitalizations. However, study results are often inconclusive, and the effects of sunlight on mental illness symptoms can vary depending on factors like duration of exposure, seasonal variations in sunlight, and patient demographics such as gender and age. Therefore, further multicenter studies are necessary to clarify these associations.

## 6. Empirical Evidence: Seasonal Trends

Considering the data presented above, it is essential to clearly identify how individual weather factors, combined to form a specific seasonal weather pattern, impact the course of mental illness and to assess whether this aligns with the concept of the immunoseasonality of mental disorders.

### 6.1. Depression

Studies have indicated that an increased frequency of depressive symptom exacerbations occurs in the fall-winter and early spring seasons [135,136,137], which coincides with conditions such as reduced sunlight, low temperatures, high relative humidity, rain and snowfall, and higher wind speeds compared to late spring and summer. This theory is also supported by evidence showing that antidepressant therapy is more frequently initiated during winter months than in summer [136].

When examining the impact of weather factors on seasonal depression exacerbations, it is essential to consider that changes in weather conditions are often associated with social, environmental, and economic factors, which may also influence mood [138,139]. A study by Brazienė et al. using a multifactorial logistic model incorporating meteorological, social, and economic factors found that the fall-winter period (November and December) was associated with a 25% increased risk of depressive symptoms in the general population. Weather factors typical of this season, such as snowfall, high relative humidity (<94%, measured two days before assessment), and increased wind speeds (4.5–6 knots), were shown to elevate the frequency of depressive symptoms, particularly in women, as measured by the Center for Epidemiologic Studies Depression Scale (CES-D 10). Additionally, temperatures above 14.2 °C (spring-summer period) were found to reduce the risk of depressive symptoms [123].

Sarran et al., who analyzed the impact of meteorological factors on depression severity among patients with seasonal affective disorder, concluded that the combination of factors such as low global radiation and sunlight duration, high humidity, and low temperatures significantly exacerbates depressive symptoms [140]. Furthermore, O’Hare et al. observed that depressive symptoms in the elderly are more pronounced during winter, with a positive correlation between rainfall in the preceding and/or current month and the severity of depressive symptoms. The authors suggest that this effect may result from reduced monthly sunlight in rainy months, as both factors may contribute to increased depression severity as assessed by CES-D scores [141].

Suicides, which may serve as an indirect indicator of depression severity within the population, are more common in the spring-summer period [142], with increased risk on days of high temperature, low relative humidity, thunderstorms, or the day following a storm [143]. Increased sunlight exposure is also notable [124], and the higher suicide rate during the spring-summer period might be attributed to an increase in motivation, partly due to increased sunlight [128] after a mood decline caused by adverse weather conditions typical of the fall-winter period.

There is also evidence suggesting a seasonal pattern in postpartum depression, with childbirth in the fall-winter period (peaking in December) associated with an increased risk of postpartum depression (PPD) [144]. Notably, women who gave birth in the last quarter of the year (fall-winter) had a higher risk of depressive symptoms both 6 weeks and even 6 months postpartum [145].

Some studies, despite demonstrating seasonality in depressive symptoms, did not find an association between increased winter symptom severity and any specific meteorological factors, suggesting that the effect may result from the combined influence of multiple climatic factors as well as other non-meteorological factors [146]. Other studies have not demonstrated seasonality in depression exacerbations, or their findings were inconsistent with the immunoseasonal theory of depression. Moreover, Øverland et al., in a 2019 systematic review, questioned the existence of a general mechanism underlying the cyclical pattern of mood decline and subthreshold depressive symptoms across the year in the general population [147]. High-quality studies are needed to further examine depression seasonality and the impact of cyclical weather changes, as well as specific weather factors, on the risk of depression exacerbations.

### 6.2. Schizophrenia

In a systematic review, Rizavas et al. demonstrated that exacerbations of major psychiatric disorders, such as schizophrenia and manic episodes, are significantly more frequent in the spring and summer [148]. Although this study partially summarizes the existing research on affective disorders and schizophrenia, we will reanalyze the key issues to establish the theoretical link between seasonality and psychiatric exacerbations.

In a study by Hinterbuchinger et al. [149], an increase in hospitalizations due to schizophrenia was observed in the summer, with a low point in December. Interestingly, a second increase in hospitalizations was observed in January, alongside the summer peak. The authors suggest that light exposure, as a source of vitamin D and a regulator of melatonin, may have implications in the exacerbation of schizophrenia [150]. The study was conducted in Austria, where January typically remains the month with the lowest amount of sunlight, which theoretically correlates with higher melatonin levels [151]. Empirically, lower light levels observed during winter periods have also been shown in several other studies [109,152]. It should be noted that melatonin shifts the inflammatory response toward Th2, and it may play a role in shifting the immune response toward an anti-inflammatory state associated with schizophrenia exacerbations [153]. Additionally, in the context of viral exacerbations during the winter, animal studies have shown that melatonin can reverse the pro-inflammatory response [154,155].

On the other hand, studies that have shown a peak in exacerbations during the winter, with decreased light availability, were mostly related to first-episode schizophrenia cases [156,157]. This aligns with the pro-inflammatory nature of the first psychotic episode in schizophrenia [158]. However, another study from China [159] similar to the studies mentioned above identified peaks of exacerbations in both summer and winter in the context of initial hospitalizations, suggesting a complex mechanism that does not exclude the immunoseasonal theory.

Additionally, other studies evaluating weather changes not during seasons but on a daily basis found no association between hospitalizations due to psychosis and the weather [160]. This suggests that, consistent with the theory, the changes are complex, requiring a characteristic seasonal pattern rather than a short-term shift. A review by Jahan et al. showed, in line with our considerations, that the peak of schizophrenia exacerbations typically occurs in summer and, in some cases, also in winter [161].

It is also worth noting that in terms of seasonal patterns, some studies do not support the immunoseasonal theory [162]. Another study, which showed an increased frequency of hospitalizations for schizophrenia in the summer, emphasizes that the overall interpretation of the season as a risk factor for psychiatric disorders is more appropriate, which contrasts with the proposed theory [163].

In analyzing the studies, a trend of increased seasonal hospitalization is notable, especially in locations with a clearer seasonal differentiation and where conditions are conducive to theoretical mechanisms of the immunoseasonal theory, as seen in Scotland [164]. Previous reviews and studies support the view of increased hospitalizations and exacerbations of schizophrenia in the summer period, with many studies also indicating a secondary peak in winter. Among specific weather factors, reduced sunlight is most frequently attributed significant importance in the literature.

### 6.3. Bipolar Disorder

The study by Amr and Volpe indicated that manic episodes peak during the summer in Egypt and, moreover, were positively correlated with temperature and brightness indices [162], unlike what has been reported for schizophrenia. It is worth noting that this data is from a country with a different seasonal pattern, where high summer temperatures may be perceived differently than winter temperatures, as discussed earlier. This could also explain results from other studies that have found an association between mania-related hospitalizations and cloud cover [109]. Interestingly, data from a subtropical climate also suggest a broader seasonal influence, with seasonal patterns typically noted for mania [165], although this data came from only one center. The systematic review by Geoffroy et al. also points to the seasonal patterns in bipolar disorder, with peaks in mania occurring in summer and early autumn, while depressive episodes tend to peak in winter [166].

Ultimately, temperature remains one of the primary factors [167] that may exacerbate the course of bipolar disorder, which may be due to interactions of temperature with humidity and the transfer of pathogens and allergens [89]. Some data did not find seasonality in bipolar disorder; however, the first admission for a depressive episode compared to a manic episode occurred significantly more often in autumn [168]. Additionally, factors such as age and sex may influence the seasonal exacerbation of mania [169].

In summary, current studies clearly indicate a pattern of more frequent hospitalizations for mania in the summer and autumn [166,170]. Expected conditions for mania during this period include higher humidity, high temperatures, and increased sunlight exposure; however, data on sunlight remain inconsistent.

### 6.4. Others—ASD, PTSD, GAD

To the authors’ knowledge, no studies have been found that assess GAD or PTSD in relation to seasonality. In the case of ASD, current data focuses on air pollution and an observed correlation with increased ASD incidence in children of mothers who became pregnant during the winter. Additionally, there are reports of ASD symptom exacerbation in cases of higher temperatures; however, data on autism remains very limited [171,172,173].

### 6.5. Season of Birth and Its Long-Term Impact on Psychiatric Disorders

Interestingly, several studies have explored the link between birth season and the risk of developing mental disorders. A meta-analysis of the Taiwanese population found an increased prevalence of schizophrenia, bipolar disorder, and MDD among individuals born in winter, while those born in autumn exhibited a higher risk of alcohol use disorder (AUD) [174]. A broader meta-analysis by Coury et al., which examined data from 30 countries, reinforced these findings, demonstrating a higher incidence of schizophrenia among individuals born in autumn and winter, while those born in summer had a lower risk. Notably, this pattern appears only in the Northern Hemisphere [175]. Additionally, studies have reported higher levels of platelet 5-HT (serotonin) in schizophrenia patients born in winter compared to those born in spring or summer, suggesting a potential neurochemical basis for these observations [176]. As potential causes of the observed seasonality, the authors point to low autumn-winter vitamin D levels due to low sunlight exposure, as well as seasonal infections during the prenatal period, which may interfere with brain development [174,175,177]. Although research seems to confirm an increased risk of schizophrenia among people born in winter, findings on the effect of the season of birth on the risk of depression and bipolar affective disorder are not consistent. Some studies did not observe an effect of season of birth on the risk of depression in adults [178], as well as indicating that delivery in spring and summer was associated with a higher risk of recurrent depressive disorder [179] and seasonality affective disorders [180], as well as a worse prognosis in MDD [181]. Schnittker points out that differences in the risk of depression by month of birth have begun to disappear over time and have historically been associated with a variable food supply depending on the season, thus emphasising the importance of the association of intrauterine conditions and nutritional deficiencies in the mother with the risk of depression in adulthood [182]. It is worth noting, however, that most studies conducted so far have primarily relied on retrospective analyses, which may limit the quality of the findings. Further longitudinal studies on large groups of patients investigating the association of risk of mental illness with the time of year of birth are necessary. 

## 7. Conclusions

Considering the data presented, there is a clear trend, which is consistent with the assumptions of the immunoseasonal theory:

We can expect exacerbations of schizophrenia (symptoms of psychosis) under conditions of reduced sunlight and, in some studies, decreased atmospheric pressure, more likely in summer. In addition, a second pick is observed that overlaps with the pick for depression: in winter, with a likely pro-inflammatory response.

For depression, exacerbations are associated with overcast days, low light availability, colder temperatures, higher wind speeds, increased relative humidity, and precipitation, all aligning with winter conditions. These environmental factors correlate with known depressive symptom fluctuations and support the immunoseasonal model.

In contrast, mania follows a different seasonal pattern, with exacerbations peaking during the summer to early autumn. This phase is typically marked by increased temperatures and prolonged sunshine, though data on humidity remains inconsistent. These findings, however, align with the immunoseasonal theory, indicating that distinct seasonal and immune-related factors influence each mental illness.

### 7.1. One Mechanism for All Mental Health Issues?

**Figure 3:**
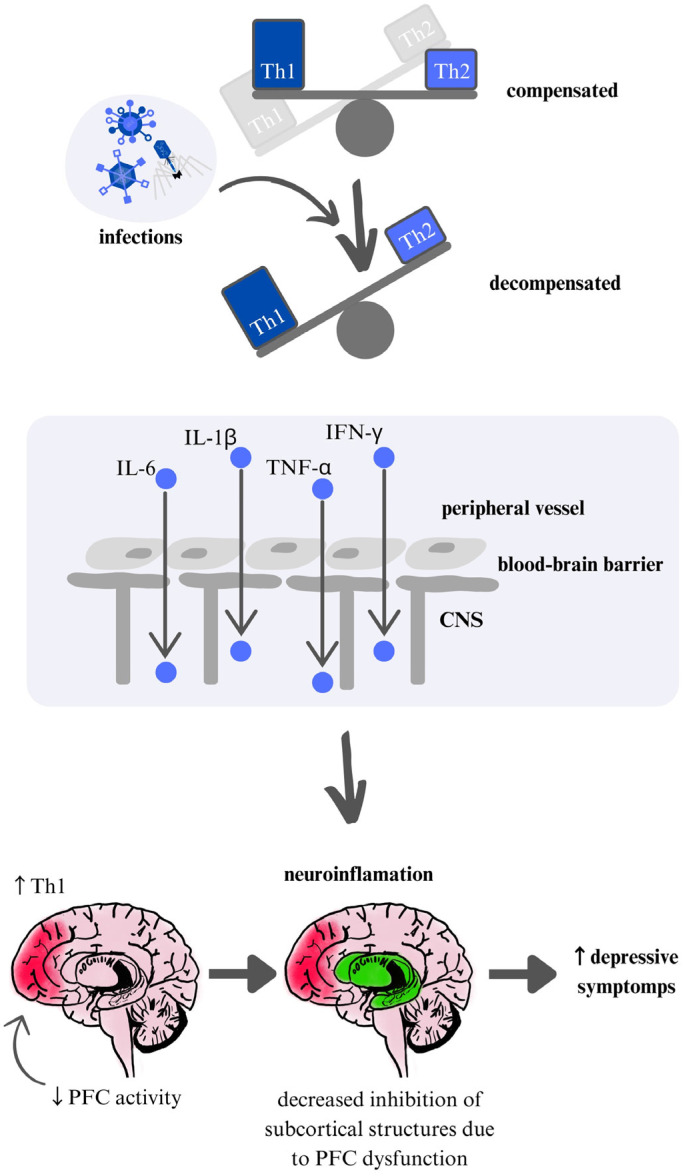
Schematic representation of the theoretical mechanism based on depression: The impact of viral infections increases the inflammatory response and decompensation. The blood-brain barrier is unsealed, resulting in neuroinflammation and loss of function of the prefrontal cortex.

Our findings suggest that seasonal fluctuations in psychiatric symptoms are associated with immune dysregulation, particularly involving shifts in pro-inflammatory and anti-inflammatory balance. Notably, increased inflammatory markers have been reported in both schizophrenia and mood disorders during seasonal exacerbations, indicating potential shared mechanisms. These results align with the immunoseasonal hypothesis, suggesting that seasonality-related immune changes may contribute to symptom modulation across different psychiatric conditions. Based on the presented data on immune markers and mechanisms, a common pathway may underlie the exacerbation of various mental disorders in winter, beginning with heightened Th1 response activation. This activation could lead to impaired prefrontal cortex function, resulting in a loss of inhibitory control over subcortical centers—a process observed in conditions like GAD and depression (see Figure 3).

In schizophrenia, however, an initial Th1 response might also trigger an excessive Th2 response, complicating the immunological profile of the disorder [65]. This dual immune activation may contribute to the complexity of interpreting psychiatric mechanisms, where the focus is on overarching disease processes rather than isolated symptoms. Such complexity helps explain the high prevalence of depressive symptoms in schizophrenia and suggests that Th1 responses may be implicated in exacerbations of these symptoms [65,183].

A broader hypothesis is that seasonal factors impact mental health holistically across diagnostic categories rather than in an isolated manner within individual patients. This could imply that seasonality functions as a risk factor for psychiatric illness overall, underscoring the need for further study into the seasonal dynamics of mental health [163].

In addition, the immunoseasonal theory also explains the phenomenon of exacerbations during the summer: during the holiday season, it typically correlates with impulsive behavior, increased risk of suicide, and also mania and psychosis, which correlates with the considerations of Dell et al. [184].

Interestingly, it is plausible to propose that the immunoseasonal theory could also provide a general framework for understanding functional changes in the suprachiasmatic nucleus (SCN), the primary biological clock of the body [185]. These alterations in SCN activity may further extend the perspective previously introduced by the Circadian Rhythm Hypothesis of Depression.

The SCN is an autonomous structure that primarily receives input from the retina (as proposed in the circadian rhythm theory) and communicates with the brainstem and hypothalamus to regulate circadian rhythms [186]. The function of the prefrontal cortex (PFC), a key regulatory center overseeing structures such as the hypothalamus and hippocampus, is impaired in depression [187]. The immunoseasonal theory attributes this dysfunction to a predominance of pro-inflammatory over anti-inflammatory responses triggered by seasonal weather changes. In general, low immunity increases the chances of disturbing the circadian rhythm [188].

A weakened DLPFC may lead to excessive activation of the HPA axis through increased corticotropin-releasing hormone (CRH) secretion in the hypothalamus [189]. Consequently, this results in elevated cortisol levels, which disrupt SCN function [186,188]. Additionally, the hippocampus—a crucial structure for encoding time and context and one of the key regions implicated in depression—remains under PFC regulation [190]. Dysfunction of the PFC may, therefore, parallel hippocampal impairments [191].

Another critical component is the locus coeruleus and the raphe nucleus, which respectively supply norepinephrine and serotonin to the SCN and are under PFC control [192,193]. PFC dysfunction may lead to a decrease in serotonin availability to the SCN, impairing its ability to synchronize with the light-dark cycle, a phenomenon observed in seasonal depression [194]. Conversely, in the case of locus coeruleus dysfunction due to PFC impairment, its hyperactivity [195] may contribute to insomnia and sleep fragmentation—both commonly observed in depression.

In the context of discussing a shared mechanism and etiology, it is also important to highlight that the influence of weather and seasonality may represent just one of many mechanisms associated with mental disorders, with its significance varying between individual patients. A valuable example in understanding this issue is the review by Favaretto et al., which explored the role of temperament in affective disorders [196,197]. Temperaments regulate emotional responses and may mediate the effect of weather and seasonality on patients’ symptoms and behaviors. Variability among different temperament types may determine whether only individuals with a specific predisposition (a given temperament) respond to sudden weather changes or prolonged seasonal shifts. For example, in the case of a cyclothymic temperament, weather-related fluctuations may be more pronounced, whereas in a hyperthymic temperament, they may be less significant. Incorporating temperament into the analysis of future studies could help explain why seasonal patterns vary among individuals with the same psychiatric disorder and enhance translational opportunities for personalized treatment approaches tailored to individual vulnerabilities. For example, a clinician working with a patient could use an assessment of their seasonal history and temperament to anticipate potential periods of exacerbation and proactively intensify care accordingly. Additionally, identifying a patient’s Th1/Th2 profile could serve as a valuable clinical indicator for individualization and predicting potential periods of disease exacerbation. If the theory proves to be valid or a specific disease pattern is identified, it may be possible, in addition to eliminating potential stressors (such as allergies or immune suppression), to consider the use of low doses of anti-inflammatory and anti-allergic medications as timing treatments for seasonal risk periods. However, we emphasize that at this stage, these considerations remain hypothetical and require further research.

### 7.2. Future Directions

It should also be noted that the relationship between weather and mental health is itself a difficult issue to research: studies to date are subject to a number of limitations, due to the fact that they come from retrospective studies, and the exclusion of other factors playing a role such as the seasonal occurrence of events with the potential to exacerbate mental illness (such as holidays), or place of residence, living situation. The need for further research should be emphasized [147]. Another challenge is the assessment of effect size in the context of the collected studies. A recent systematic review by Rizavas et al. highlighted significant heterogeneity among studies evaluating the seasonality of psychiatric disorder exacerbations, which prevented the authors from conducting a meta-analysis [148]. However, the researchers noted that the majority of studies indicate a trend of worsening psychiatric disorders during the summer, particularly in cases of mania and schizophrenia.

These findings are consistent with a meta-analysis on the effects of specific weather factors [110]. Their metaanalysis shows that heatwaves (pooled RR = 1.05, 95% CI = 1.02 1.08) and extremely high temperatures (99th percentile: pooled RR = 1.18, 95% CI = 1.08 1.29) were associated with an increased risk of disorders such as schizophrenia and mood disorders. Given these findings, assessing the overall effect of seasonal changes on the course of psychiatric disorders remains challenging and requires further investigation. Future research should aim to develop standardized methodologies for evaluating the effect of seasonality, including consistent definitions of seasonal patterns, harmonized study designs, and uniform statistical approaches to facilitate cross-study comparisons. 

To validate the immunoseasonal mechanism, comprehensive studies are essential. Observational and retrospective analyses should aim to confirm exacerbation patterns and further characterize patient symptoms, as well as conduct rigorous laboratory assessments. Additionally, analyzing diurnal rhythms would be crucial to evaluate their role in brain function and broader clinical implications, as highlighted by Zhang and Volkow [198]. Epidemiological and social characteristics, such as major holidays and other factors that could distort the effect of weather, should also be considered. Economic factors like place of residence, vegetation depletion, climate, as well as patient demographics like age and gender (see Table 1) must be accounted for to ensure data accuracy.

Apart from further retrospective studies, an analytical, clinic-based, and multivariate cross-sectional study should be conducted. To substantiate this theory, a wide panel of inflammatory markers should be evaluated, encompassing both pro- and anti-inflammatory cytokines and assessing viral infection markers (through specific PCR tests, indirect markers like IFN-α and IFN-γ; [9,57] as well as general allergic responses (e.g., non-specific IgE, tryptase, blood smear). Key biomarkers to be analyzed include IFN-α, IFN-γ, IL-1β, IL-6, TNF-α, CRP, IL-4, IL-10, and IL-17, along with interleukin ratios to determine immune response dominance [199], including IL-6/IL-10. From a practical perspective, inflammatory markers should be correlated with the patient’s clinical status, ideally using standardized scales such as the Montgomery-Åsberg Depression Rating Scale [200] and the Positive and Negative Syndrome [201] Scale. From an interview standpoint, it is crucial to assess when the deterioration occurred (as symptom onset often precedes hospital admission) to accurately determine the meteorological conditions relevant to the appropriate time period. Such an approach would allow for a preliminary demonstration of either an association or a lack thereof between clinical status, immunological markers, and weather conditions. A particularly beneficial strategy would involve conducting cohort studies, in which the procedure would be similar to the one described above but also repeated during remission periods.

The next step, if the analysis reveals preliminary associations, would be an attempt to identify potential triggers of exacerbations by assessing virological and allergological panels and evaluating their distribution in comparison to patients who do not exhibit a seasonal course of illness.

Further, the most robust approach would involve imaging studies to verify physiological differences between remission and exacerbation states correlated with seasonal fluctuations, providing deeper insights into immune involvement across different phases of mental health conditions.

## Figures and Tables

**Table 1:** Suggested questions and information that can help in an interview directed at seasonality.

General Information	Preliminary Questions	Masking Seasonality	Symptom Characteristics Considering Seasonality and Individual Weather Factors
Age	Is the course of the illness fluctuating so far?	Recent events, typically observed in a given season and associated with social impact	General characteristics of the exacerbation and course of the illness: Intensification, symptom alleviation, treatment attempts
Gender	How many previous hospitalizations were there and in which season or time of year?	Since when and what medications or substances are being taken?	What is the patient’s daily rhythm?
Reason for hospitalization	Has a difference in clinical presentation been observed in different seasons?	Is the patient’s schedule regular, e.g., 8-h workdays, or variable?	Does the patient take vitamin D?
Diagnosed Condition	Is there a specific season that seems particularly difficult for the patient?	Does the patient actively use other forms of treatment?	How does the patient assess their sleep? How many hours do they sleep? Do they work remotely or outdoors?
	Are there any global events (e.g., war) that might have been overlooked but significantly impact the patient?	Recent Endocrine Problems: menopause, PMS, thyroid issues, and others. Other chronic illnesses.	Has the patient used fototherapy, and with what result?
		What conditions does the patient experience at home and work? (cold, humidity, wind, light exposure)	How does the patient assess the air quality where they live?
		Has the patient recently returned from traveling to another country or climate zone? Have they recently spent time near water?	How does the patient assess their immunity?
		What is the patient’s level of physical activity?	Does the patient suffer from allergies or allergic diseases
			Does the patient have parasites?
			What is the patient’s residence (considering distance to water bodies, pollution, presence of allergens, etc.)
